# Aquatic model for engine oil degradation by rhamnolipid producing *Nocardiopsis* VITSISB

**DOI:** 10.1007/s13205-014-0199-8

**Published:** 2014-03-26

**Authors:** Suki Roy, Shreta Chandni, Ishita Das, Loganathan Karthik, Gaurav Kumar, Kokati Venkata Bhaskara Rao

**Affiliations:** 1 Molecular and Microbiology Research Laboratory, School of Bio Sciences and Technology, VIT University, Vellore, 632014 Tamil Nadu India; 2 Department of Biotechnology, Shri JJT University, Jhunjhunu, Rajasthan India

**Keywords:** Actinobacteria, Bioremediation, Biosurfactant, Rhamnolipid

## Abstract

The present study was focused on isolation, screening, characterization and application of biosurfactant producing marine actinobacteria. Twenty actinobacteria were isolated from marine water sample and were primarily screened for biosurfactant production using hemolytic activity method. Among the 20 isolates, six showed positive result for hemolytic activity and those were taken for further secondary screening tests such as oil collapse method, oil spreading method and emulsification method. From the results of secondary screening analysis, two isolates (SIS-3 and SIS-20) were selected and further used to carry out biosurfactant characterization test such as pH, density, surface tension and viscosity determination. Comparing biosurfactant characterization results, SIS-3 was chosen for further analysis and application. FT-IR and GC–MS were carried out for analysis of biosurfactant from isolate SIS-3 and the compound detected was rhamnolipid. The isolate (SIS-3) was identified as *Nocardiopsis* using 16S rRNA gene sequencing and named as ‘*Nocardiopsis* VITSISB’ (KC958579) which was further applied for immobilizing whole cells for engine oil degradation by constructing an aquatic model and using natural products such as soybean meal, sugarcane juice as nutrient source. The oil was efficiently degraded by rhamnolipid producing *Nocardiopsis* VITSISB (KC958579) within 25 days which indicated that the strain can act as a natural candidate for the bioremediation of oil spill in ocean.

## Introduction

Disasters make headlines, but a hundreds and million gallon of oil quietly ends up in the sea every year which is not considered seriously. It has been analyzed that a large percentage of ocean pollution occurs due to oil leakage in marine water. When it comes to oil pollution, ocean is the worst sufferer in terms of loss of aquatic life (Murali et al. 2010). There are many incidents that have occurred worldwide. One of the recent oil spill incident reported by “NY Daily News”, that in 27 July 2013, oil spilling occurred in the sea of Rayong near eastern Thailand due to the leakage of a pipe line of Oil and Natural Gas Company “Petroleum Authority Of Thailand Public Limited Company” (PTT Plc), buried deep into the ocean up to 20 km which spread nearly 50,000 tons of oil up to 984 feet near the eastern cost of Thailand. It caused lot of damage to the environment. Apart from this, many other incidences had occurred in all over the world due to many different causes such as oil leaked into the sea, accident of oil transporting ships from one place to another, which also affect valuable resources of the natural environment. Research is going on to find out the relevant solutions with respect to factors such as eco friendly and cost effective methods.

Biodegradation of oil by biosurfactant producing strain plays an important role in restoring oil-polluted environments and habitats. Some microorganisms can produce amphoteric surface-active-agents which emulsify hydrocarbons in a growth medium and reduce the surface and interfacial tensions, which increase the solubility, and emulsification of the two immiscible phases also provides an insoluble substrate for microorganisms (Banat et al. 2000). These are known as biosurfactants. These are a structurally diverse group of surface-active molecules which are eco-friendly. It has been characterized that, biosurfactants having advantages of low toxicity, high biodegradability, better environmental compatibility, high foaming capability, higher selectivity, specific activity at extreme temperature, pH, salinity, can be analyzed for its applications for the specified issue.

Biosurfactants have been broadly used in environmental protection purpose; such methods are enhanced oil recovery (EOR), control of oil spills, detoxification of industrial effluents and soil contaminated with oil. Chemical dispersants that are mainly used to dissipate oil slicks lead to the increase in toxic hydrocarbon levels in fish by a factor of up to 100 and may kill fish eggs. Dispersed oil droplets infiltrate into deeper water and can lethally contaminate coral. Research indicates that some dispersants are poisonous to corals. A 2012 study found that Corexit dispersant had increased the toxicity of oil by up to 52 times. On the other hand, biosurfactants have an amplified adaptability in contrast to numerous chemical surfactants; therefore it may be appropriate for bioremediation (Borjana et al. 2001).

There is a dependence of biodegradation of a given hydrocarbon on its dispersion site (Mattei et al. 1986). However, minute information on either biosurfactants active in saline or biosurfactant produced by marine microorganisms has been reported so far. Marine biosurfactants produced by some marine microorganisms have been rewarded more interest, particularly for the bioremediation of the ocean polluted by crude oil (Maneerat 2005). It is not surprising that marine actinobacteria are found to be a valuable resource since they are different from terrestrial organisms due to their survival in adverse conditions of marine environment such as alkalinity, temperature etc. Marine biotechnology has opened up unexpectedly new horizons for discovering novel organisms and trapping their potential resources such as bioactive compounds which are widely used in different applications such as pharmaceutical, textile, cosmetic industries etc. (Fiedler et al. 2005; Blunt et al. 2009). The actinobacteria are distinguished by their gram-positive character and high guanine-plus-cytosine (G + C) content in their genomes (Okami 1986).

The present study was to isolate and characterize biosurfactant producing actinobacteria from marine water and to evaluate their application in engine oil degradation.

## Materials and methods

### Medium, chemicals and engine oil

All the media and chemical ingredients used in the current experiment were bought from Hi-Medium, Mumbai, India and SRL Pvt. Ltd., Mumbai, India. Engine oil used in this study was obtained from local automobile workshop (Vellore, India) and was sterilized by filtration.

### Isolation of marine actinobacteria

The marine water sample was collected from Marina beach (13.05°N, 80.28°E), Chennai, Tamil Nadu, India. A tenfold serial dilution of the sample was prepared. 100 μl of serially diluted sample was inoculated into starch casein agar medium (pH 7.2) prepared with 50 % seawater to sustain the growth of actinobacteria. To avoid the growth of fungal and bacterial contaminant, potassium dichromate (50 μg ml^−1^) and nalidixic acid (15 μg ml^−1^) were supplemented. The plates were incubated at room temperature (28 °C) and monitored periodically over 3 months for actinomycetes growth. The pure isolates of the actinobacteria were transferred to ISP2 (Isolation streptomyces project medium No. 2) slants and preserved at 4 ± 2 °C (Karthik et al. 2010; Kathiresan et al. 2005).

### Primary screening method

Biosurfactant producing marine actinobacteria were primarily screened using hemolytic assay. All the isolates were streaked into blood agar plate and incubated at 37 °C for 7 days. The appearance of clear zone indicated the production of biosurfactant. The results were interpreted as ‘*γ*’ for no hemolysis, ‘*α*’ for incomplete hemolysis and ‘*β*’ for complete hemolysis (Mulligan and Gibbs 1989).

### Production media for biosurfactant

To select an appropriate growth media for biosurfactant producing actinobacteria, two different media were used. These are SS medium (soluble starch 25 g/l, glucose 10 g/l, yeast extract 2 g/l, calcium carbonate 3 g/l, trace salt solution 1 ml (FeSO_4_, CuSO_4_, ZnSO_4_ and MnCl_2_) and SS medium with oil (SS Medium with 0.5 % oil). One loop full culture of the isolates were inoculated into two media and incubated at 37 °C for 7 days. After 7 days, emulsification index was calculated for each medium.

### Secondary screening method

Biosurfactant producing marine actinobacteria were screened by drop collapsing method, oil spreading method and emulsification index. Sodium lauryl sulphate was taken as positive control for all the methods (Chandran and Das 2010).

### Drop collapse method

In drop collapse method, 2 μl of mineral oil was added to 96-well microtiter plate and 5 μl of the culture was added to the surface of the oil. After 1 min, the observation of shape of the drop on the surface of the oil was done. The interpretation of result was ‘+’ to ‘++’ corresponding to partial to complete spreading on the oil surface. Those cultures that gave rounded drops were scored as negative ‘−’ indicative of the lack of biosurfactant production (Bodour and Miller-Maier 1998).

### Oil spread method

In Petri plate, 40 ml of distilled water was added followed by 10 μl of engine oil on the surface of the water. 10 μl of culture was then added to the surface of oil. The diameter of the clearing zone on the oil surface and correlation of surfactant activity were measured (Walter et al. 2000).

### Emulsification index (*E*_24_ %)

Emulsification activity was measured by calculating *E*_24_ described by Cooper and Goldenberg (1987). Kerosene was added to culture supernatant (1:2 v/v), mixed in a high speed vortex for 2 min and allowed to stand for 24 h. After 24 h, measurement of height of the stable emulsion layer was executed. The emulsion index *E*_24_ is calculated as the ratio of the height of the emulsion layer and the total height of liquid.

1 $$E_{24}=\left(\text{total height of the emulsified layer / total height of the liquid layer}\right)\times 100\;\%$$

### Production and extraction of biosurfactant

Biosurfactant production was carried out in SS Medium with 0.5 % oil. One loop full culture of the isolates were inoculated into media and incubated in a shaker at 120 rpm for a period of 7 days at 37 °C. Centrifugation of the culture broth was carried out at 10,000 rpm for 10 min followed by extraction of solvents with chloroform and methanol (2:1 v/v). The solvent removal was done by rotary evaporation and biosurfactant was obtained as the resultant residue (Chandran and Das 2010).

### Biosurfactant characterization

Biosurfactant characterization was conducted by determination of pH, density, surface tension and viscosity. In all the cases, sodium lauryl sulphate was taken as positive control and distilled water as negative control.

### pH and density determination

The pH and density of the biosurfactant solution (1 mg/ml) was determined using pH meter and hydrometer, respectively.

### Surface tension measurement

The capillary rise method was used to determine the surface tension of the culture free supernatant. A small bore capillary was inserted into the liquid whose surface tension was to be determined. The liquid level raised to a certain height in the tube was proportionally varying with the surface tension. The surface tension was calculated using the following formula (Harkins and Jordan 1991):

2 γ=dghr/2

where, *γ* is surface tension, *d* is the density of the fluid, *g* is the acceleration due to gravity, *h* is the height of liquid rising and *r* is the radius of capillary’s bore.

### Viscosity determination

High viscosity of the oil limits its mobility. So, decrease in the viscosity of heavy oil is a way to improve oil recovery. The culture-free supernatant was used to determine the viscosity of the biosurfactant with the help of Ostwald viscometer. Distilled water was used as reference fluid. The formula used to determine viscosity was:

3 $$\eta_{\text{s}}=\left(\eta_{\text{w}}t_{\text{s}}\rho_{\text{s}}\right)/t_{\text{w}}\rho_{\text{w}}$$

$\eta_{\text{s}}$ is the viscosity of sample, $\eta_{\text{w}}$ is the viscosity of water, *t*_s_ and *t*_w_ are the time taken by sample and water, respectively, *ρ*_s_ and *ρ*_w_ are the density of sample and water, respectively (Cooper et al. 2013).

## Analysis of biosurfactant

### Orcinol assay

The nature of the biosurfactant was determined using orcinol assay. In this assay, 100 μl of cell-free supernatant was added to 900 μl of orcinol reagent and incubated at 80 °C for 30 min. Production of a blue-green color determined the presence of glycolipid in the supernatant. The mixture was allowed to come down to room temperature. The absorbance was measured at 421 nm (Harkins et al. 2010).

### Stability analysis

To evaluate the temperature effect, 10 ml of the culture-free supernatant was exposed at 5, 50, 70 and 100 °C for 1 h and cooled to room temperature. To examine the pH effect, the pH of the culture supernatant was adjusted to 2.0, 4.0, 8.0 and 12.0. The effect of sodium chloride concentration was detected by adding sodium chloride into the medium to achieve the concentration of 2.0, 4.0, 7.0 and 10.0 % (w/v). Stability study was conducted by surface tension and emulsification index determination.

### Fourier transforms infrared spectroscopy (FT-IR) analysis

One milligram of extracted biosurfactant was mixed with 20 mg of potassium bromide and pressed to obtain translucent pellets. The infrared spectra were performed on Mattson 1,000 FT-England FTIR system within the range of 500–4,000 cm^−1^ wave number (Chander et al. 2012).

### Gas chromatography–mass spectrometry (GC–MS) analysis

The chloroform:methanol extract of SIS-3 was analyzed by gas chromatography–mass spectrometry (GC–MS) using a Thermo GC—Trace Ultra VER: 5.0, Thermo MS DSQ II with a Elite-5MS (30.0 m, 0.25 mm ID, 250 μm df). The temperature of the column was programmed from initial temperature 60 °C for 2 min, ramp 10 °C/min to 300 °C with a holding of 6 min, and the injector or detector temperature for the analysis was about 250 °C. Helium was used as the carrier gas at a flow rate of 1.0 ml/min. The identification of the chemical constituents was based on correlation with the existing mass spectra data with those obtained from the Wiley8.LIB and NIST08.LIB library spectrum provided by the software on a GC–MS system (Arino et al. 1996).

### Strain identification

The potent strain showing significant biosurfactant activity was molecularly characterized with 16S rRNA gene sequencing. The actinobacterial DNA was isolated by Bacterial DNA Mini Spin Kit (2 μl) (Amnion, India) and PCR amplification was done using an initial denaturation step at 94 °C for 5 min followed by 35 cycle of 1 min at 94 °C, 30 s at 55 °C and 1:30 min at 72 °C and final extension at 72 °C for 5 min cooled at 4 °C, the Taq polymerase was used as 10× AMTaq Polymerase buffer: 5 μl AMTaq, Polymerase enzyme: 3 U and PCR grade water to make the volume up to 50 μl (Amnion, India) as per user manual (Saiki et al. 1985). The forward and reverse primers used are 5′-CWG RCC TAN CAC ATG SAA GTC-3′ (100 ng) and 5′-GRC GGW GTG TAC NAG GC-3′ (100 ng), respectively. The resemblance or homogeneity of the16S rRNA partial gene sequence was analyzed with the analogous sequences already present in the records bank-National Center for Biotechnology Information (NCBI) via BLAST search. The applied DNA sequences were allied and phylogenetic tree was created with the function of neighbor-joining technique, the used online software is known as ClustalW (Saitou and Nei 1987). The topologies of the resulted tree were calculated through bootstrap analyses of the neighbor-joining technique based on 1,000 re-samplings. The phylogenetic analysis was conceded using the TREEVIEW software (version 1.3.3).

### Immobilization of whole cells of SIS-3 in calcium alginate beads

Immobilization was carried by suspending 100 ml of cultures in sodium alginate solution (2 % w/v). The mixture obtained was added dropwise through a syringe into calcium chloride solution (3.5 % w/v). Beads were formed when alginate got solidified upon contact with calcium chloride_,_ entrapping the cells. The spherical beads (~2 mm diameter) got hardened in 30 min and then were washed with distilled water to eliminate excess calcium ions and cells (Taha et al. 2013).

### Degradation of engine oil by designing aquatic model

Oil degradation ability of the biosurfactant was determined by setting up an aquatic model. It was constructed with an open glass chamber provided with 2 l of marine water, 100 ml of engine oil and limestone. To degrade the oil, whole-cell immobilization of *Nocardiopsis* VITSISB (KC958579) in calcium alginate was conducted. The immobilized cells were exposed to oil present in the aquatic model along with 100 ml of sugarcane juice and 50 mg of soya meal which acted as nutrient for the cells. The model was kept at room temperature for 25 days. Oil sample from the aquatic model was centrifuged at 5,000 rpm for 5 min. Water layer was discarded and the oil supernatant was taken. The oil was then extracted by means of chloroform (1:1) in separating funnel. Chloroform and supernatant was vortexed for 5 min and kept still for 10 min. The lower aqueous layer and top layer of chloroform with engine oil were taken out in another flask. A little amount of anhydrous sodium sulphate was used to eliminate the residual moisture. Solvent hexane was then evaporated at room temperature. The remaining oil left was then diluted with chloroform in the ratio of 1:9 (v/v) (Kumari and Abraham 2011). The oil degradation was analyzed using High Performance Liquid Chromatography (Empower Company, India). Acetonitrile:water (75:25 %) was used as mobile phase. The HPLC analysis was performed at a flow rate of 1 ml min^−1^, with UV detection at a wavelength of at 244 nm.

### Statistical analysis

All experiments were carried out in triplicates. Data are presented as mean ± standard deviation (SD). To prove significant differences, results were evaluated statistically, at 95 % confidence level (*p* < 0.05), using the Analysis of Variance (ANOVA) available in Graphpad Prism v.6.00 (La, Jolla, CA, USA).

## Results

**Fig. 1:**
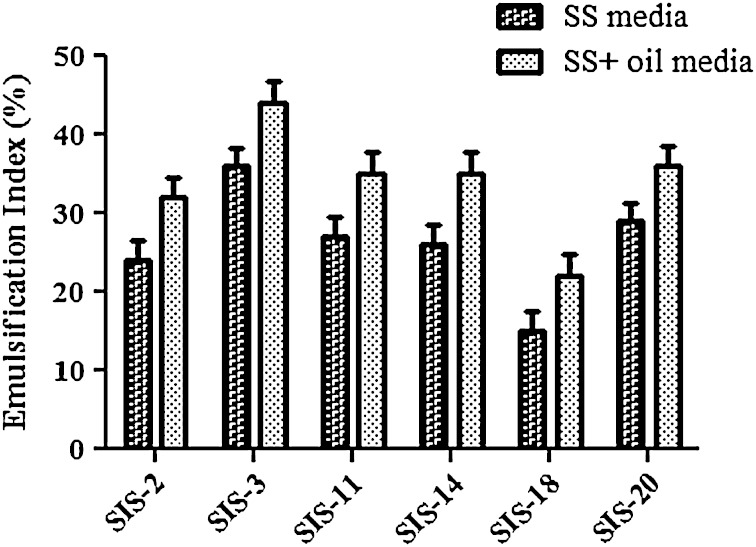
Emulsification index (*E*_24_) of the isolates in two media. Data are given as mean ± SD (*n* = 3). Results were considered significant at *p* < 0.05

**Fig. 2:**
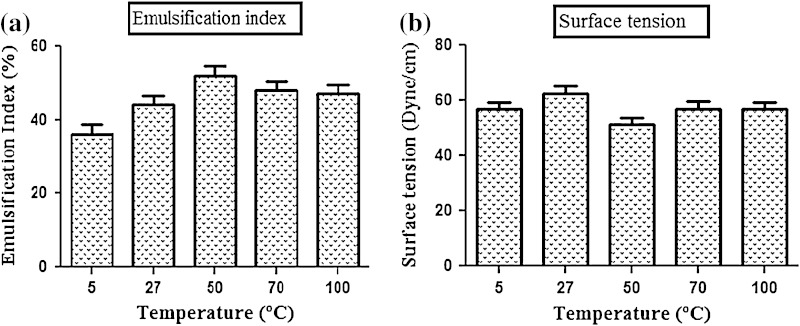
Temperature stability of biosurfactant produced by SIS-3: **a** emulsification index, **b** surface tension. Data are given as mean ± SD (*n* = 3). Data are significant where *p* < 0.05

**Fig. 3:**
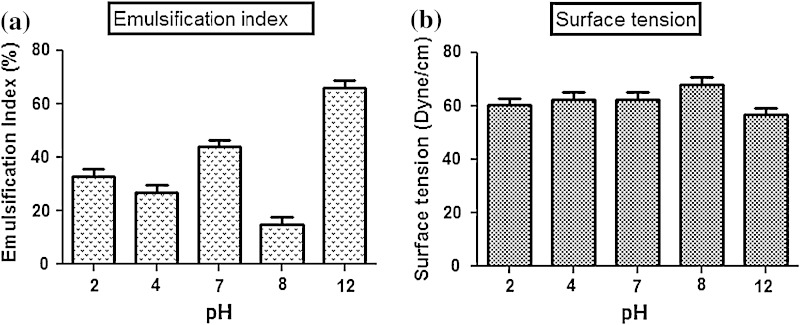
pH stability of biosurfactant produced by SIS-3: **a** emulsification index, **b** surface tension. Data are given as mean ± SD (*n* = 3). Results were considered significant at *p* < 0.05

**Fig. 4:**
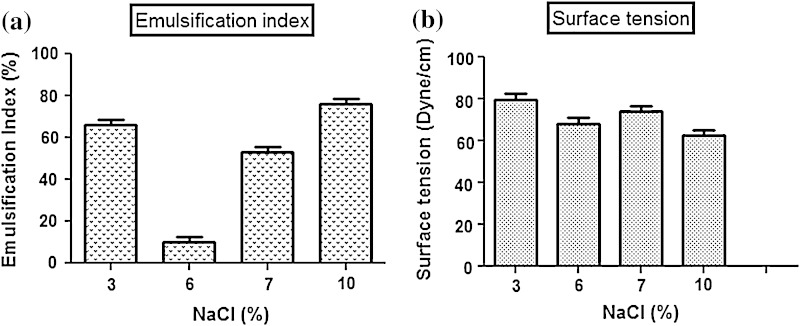
Salinity stability of biosurfactant produced by SIS-3: **a** emulsification index, **b** surface tension. Data are given as mean ± SD (*n* = 3). Results were considered significant at *p* < 0.05

**Fig. 5:**
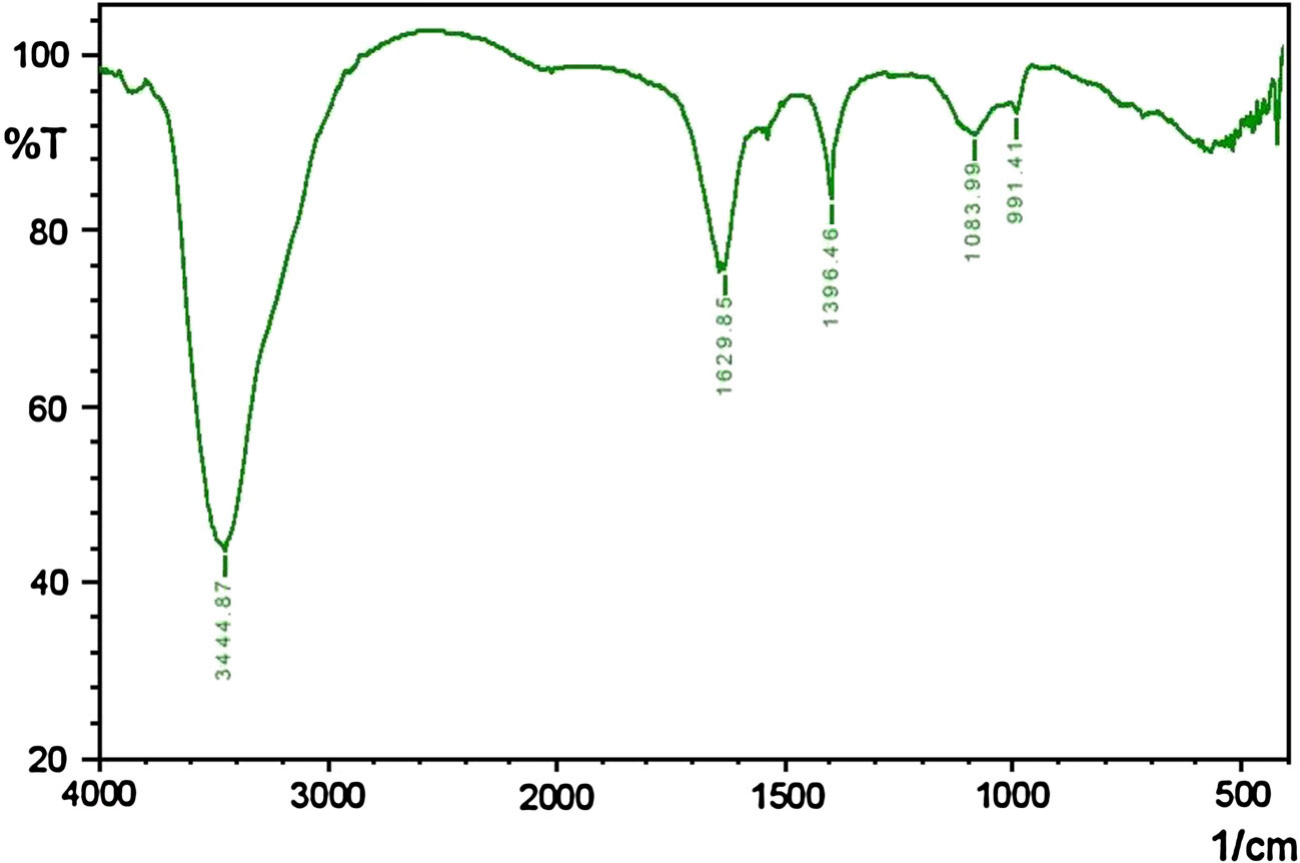
FT-IR spectra of biosurfactant produced by SIS-3

**Fig. 6:**
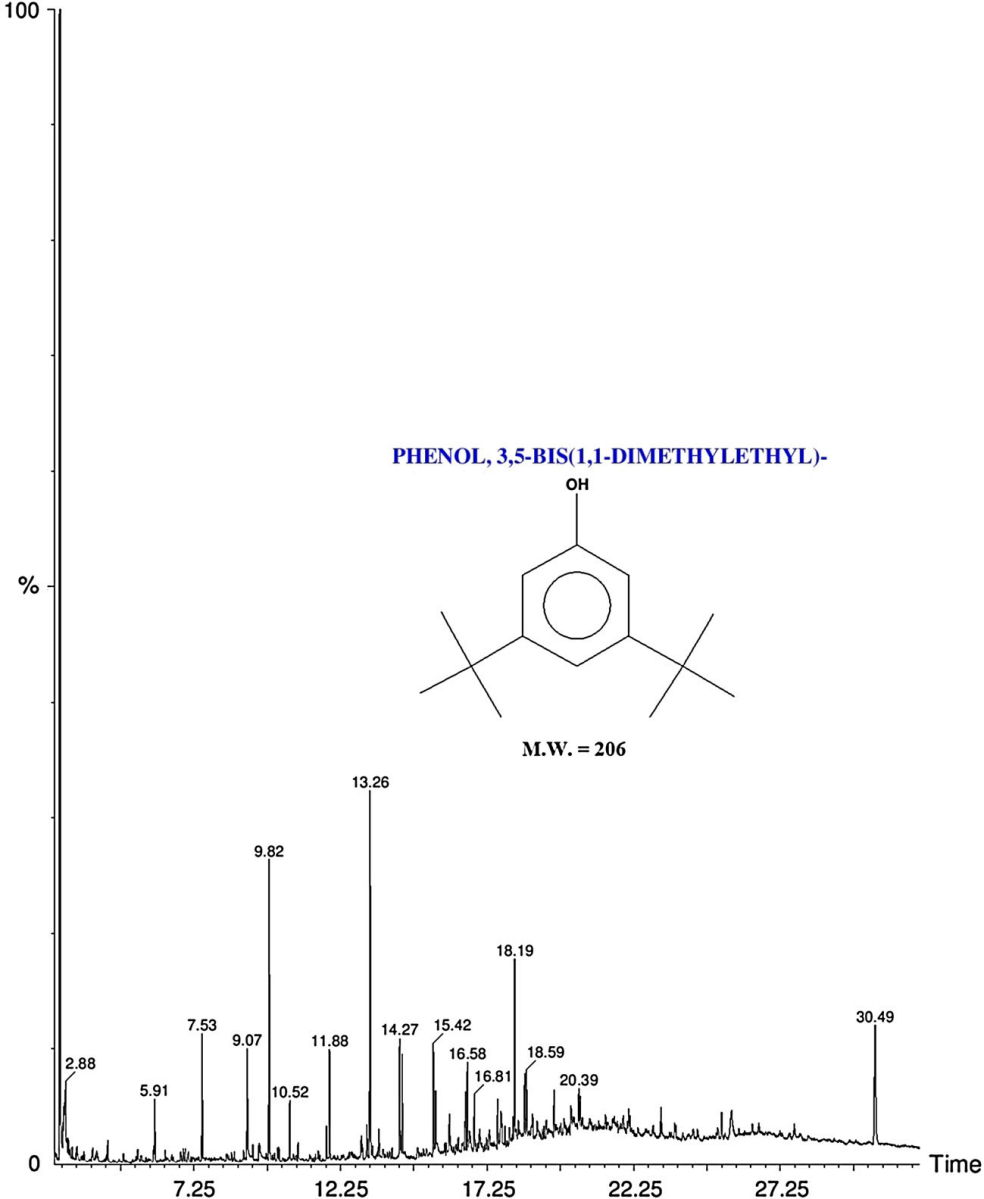
GC–MS profile of chloroform:methanol extract of SIS-3

**Fig. 7:**
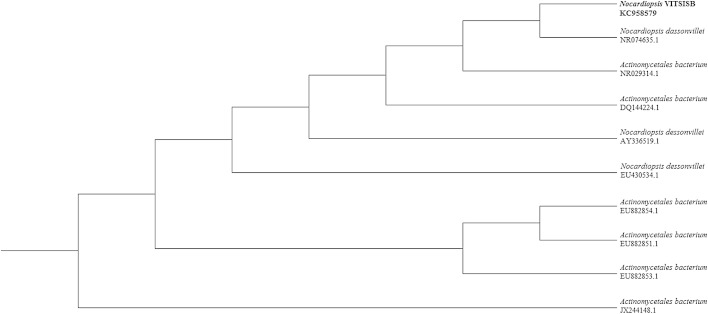
Phylogenetic tree of *Nocardiopsis* VITSISB (KC958579)

**Fig. 8:**
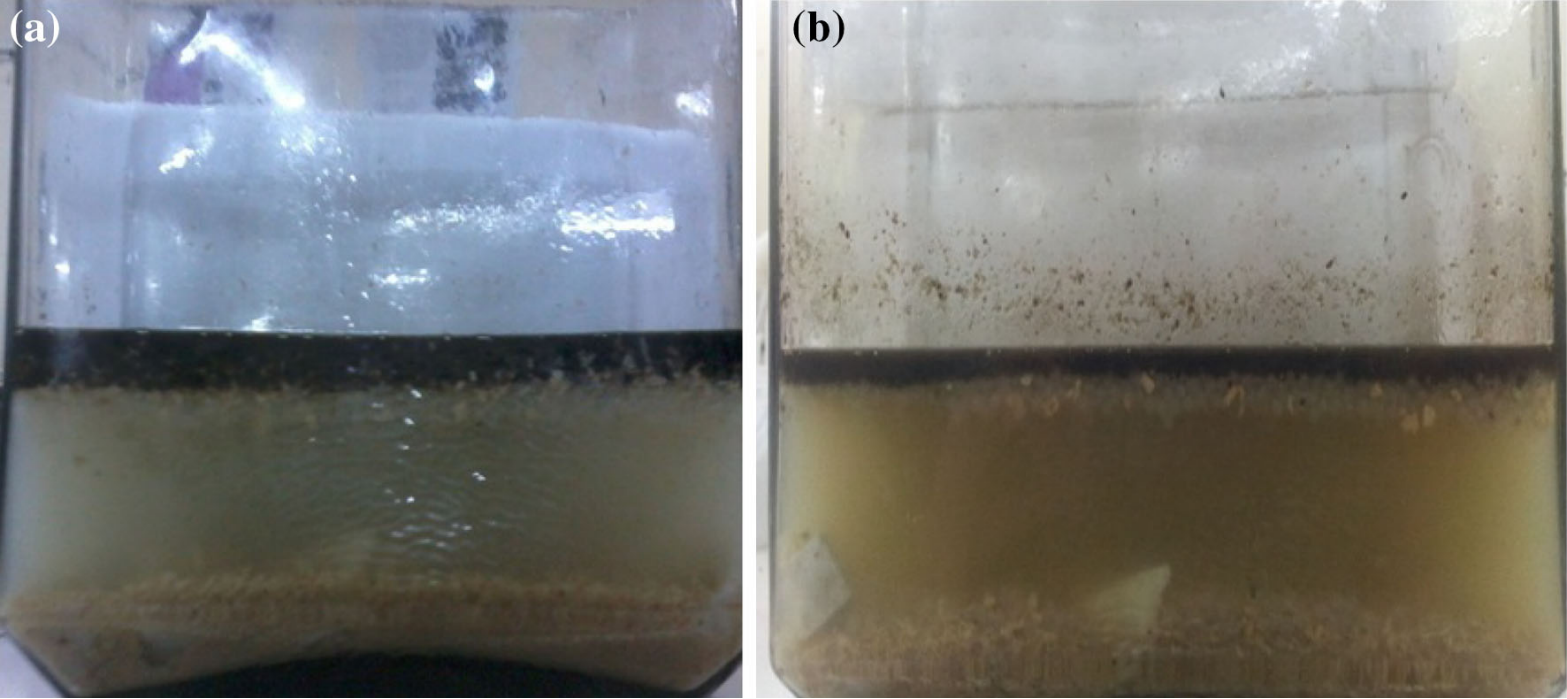
Aquatic Model **a** day 1 and **b** day 25

**Fig. 9:**
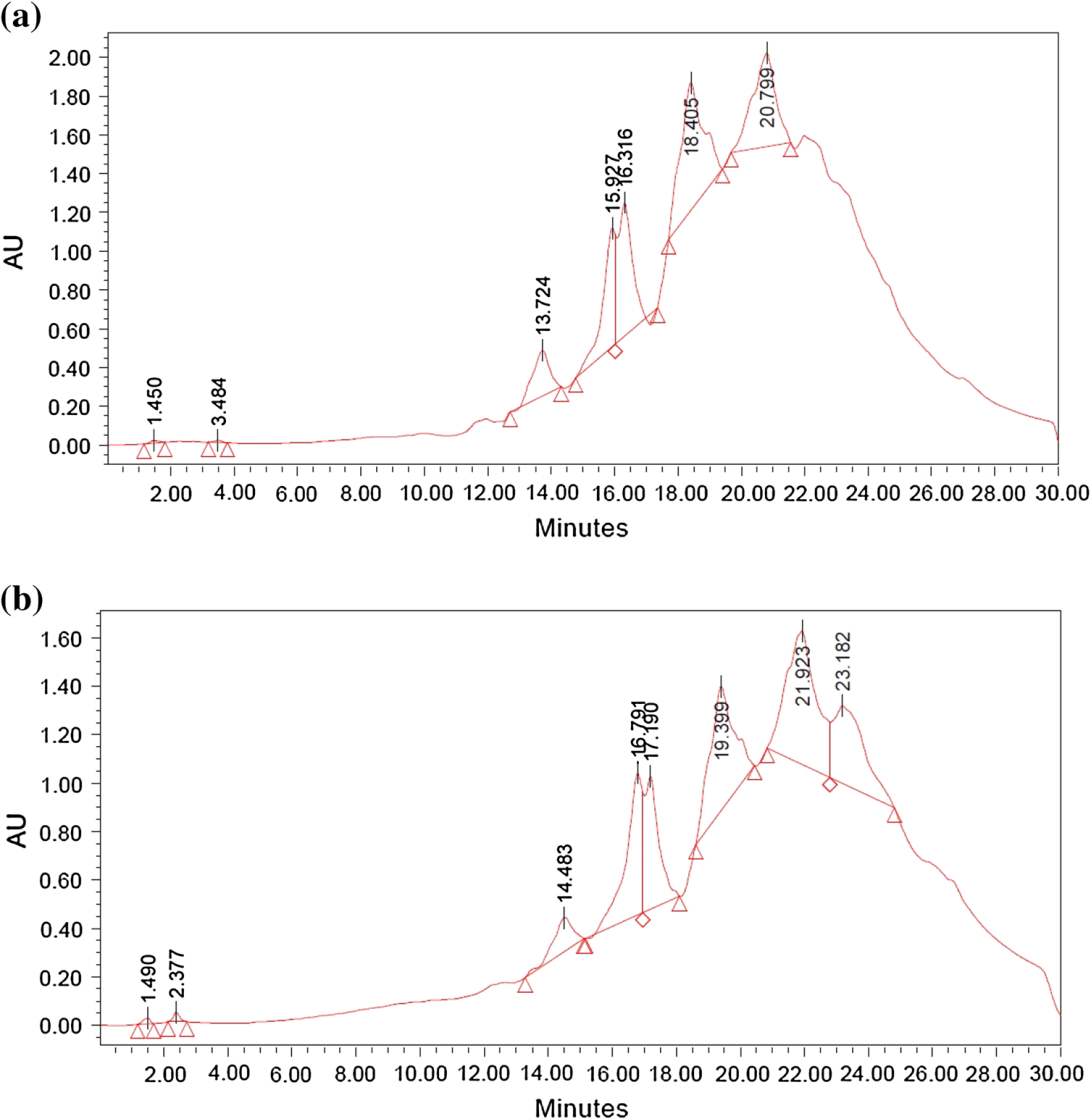
HPLC profile of engine oil: **a** before degradation, **b** after degradation (25 days)

Total 20 marine actinobacteria were isolated from marine water sample. Out of 20 isolates, six isolates showed positive result in hemolytic activity on blood agar plates. The results are presented in Table 1. Two media (SS, SS + OIL) were used for the production of biosurfactant by marine actinobacteria. The emulsification index for the isolates in two media is mentioned in Fig. 1. The result of secondary screening of biosurfactant by six isolates is provided in Table 2. Biosurfactant characterization test were conducted and the results are summarized in Table 3. The surface tension was observed as 62.808 dyne/cm for SIS-3, 62.808 dyne/cm for SIS-20, and 68.6 dyne/cm for water. With reference to biosurfactant viscosity measurement result, SIS-3 showed 10.7 × 10^−3^ poise, SIS-20 showed 12.6 × 10^−3^ poise and sodium lauryl sulphate showed 10.1 × 10^−3^ poise, so lower the viscosity more would be the positive effect on emulsion stability of biosurfactant. Density measurement result showed SIS-3 as 1.16 g/cm^3^, SIS-20 as 1.18 g/cm^3^ and water as 68.6 g/cm^3^. The orcinol assay showed positive result by formation of blue-green color. The constancy of biosurfactant produced by SIS-3 was examined over an extensive variation of temperatures.The surface tension reduction (dyne/cm) and percentage of emulsification activities were highest at the temperature of 50 °C as shown in Fig. 2. The biosurfactant showed relatively high efficiency at pH 12 where the emulsification activity showed almost 66 % emulsion and the surface tension reduced to 56.8 dyne/cm as shown in Fig. 3. The effect of sodium chloride addition on biosurfactant produced from SIS-3 was studied as shown in Fig. 4. Optimum constancy of biosurfactant was observed at 10 % NaCl concentration. At higher concentration of sodium chloride, the biosurfactant showed 76 % of the emulsification activity and more reduction in surface tension than in lower concentration of NaCl. Molecular compositions of biosurfactant produced by SIS-3 were evaluated by FT-IR as shown in Fig. 5. The broad band observed in biosurfactant was at 3,444 cm^−1^ which corresponded to the hydroxy group, H-bonded OH stretch in glycolipid. The peak at 1,689 cm^−1^ corresponded to C=O stretching of carbonyl groups which is characteristic for ester compounds. The band 1,396 cm^−1^ confirmed the presence of alkyl groups. The C–O–C stretching in the rhamnose was found at 1,083 cm^−1^ and the stretching of aromatic C–H in-plane bend occurred at 991 cm^−1^. The GC–MS analysis of chloroform:methanol extracts of SIS-3 is shown in Fig. 6. The results showed that the major chemical constituent of chloroform:methanol extracts of SIS-3 was phenol, 3,5-bis(1,1-dimethylethyl)- by comparison of mass spectral data and retention times. The other major constituents present in the extract were benzene, 1,3-bis(1,1-dimethylethyl),dibutyl phthalate,2-tert-butyl-4,6-bis(3,5-di-tert-butyl-4 hydroxybenzyl) phenol and dodecane, 1-fluoro. The major chemical constituent phenol,3,5-bis(1,1-dimethylethyl) was responsible for surfactant activity. The molecular weight of phenol, 3,5-bis(1,1-dimethylethyl) (C_14_H_22_O) was 206. The taxonomic position of *Nocardiopsis* VITSISB (KC958579) was determined based on 16S rRNA partial gene sequencing. A phylogenetic tree was constructed based on the neighbor-joining method using Treeview software as shown in Fig. 7. The oil layer in the aquatic model was reduced after duration of 25 days as shown in Fig. 8. HPLC Chromatogram of degraded oil showed more number of peaks as compared to control engine oil as shown in Fig. 9.

**Table 1:** Haemolysis assay of 20 isolates of marine actinobacteria

Isolate name	Haemolysis activity	Size of zone of clearance (mm)
SIS-1	α	9 ± 0.5
SIS-2	β	18 ± 0.3
SIS-3	β	15 ± 0.5
SIS-4	α	8 ± 0.6
SIS-5	α	6 ± 0.6
SIS-6	α	5 ± 0.3
SIS-7	β	5 ± 0.6
SIS-8	α	6 ± 0.5
SIS-9	γ	
SIS-10	γ	
SIS-11	β	17 ± 0.6
SIS-12	α	3 ± 0.5
SIS-13	γ	-
SIS-14	α	16 ± 0.6
SIS-15	α	7 ± 0.5
SIS-16	α	4 ± 0.5
SIS-17	γ	-
SIS-18	β	16 ± 0.3
SIS-19	α	10 ± 0.2
SIS-20	α	13 ± 0.4

**Table 2:** Secondary screening of biosurfactant produced by 6 isolates

Isolate name	Oil collapse method	Oil spread method Size of zone (mm)	Emulsification index (%)
SIS-2	+^a^	62 ± 0.2	32 ± 0.4
SIS-3	+	74 ± 0.5	44 ± 0.2
SIS-11	-^c^	64 ± 0.3	35 ± 0.3
SIS-14	+	75 ± 0.2	35 ± 0.1
SIS-18	+	52 ± 0.4	22 ± 0.2
SIS-20 SLS	++^b^ ++	68 ± 0.3 83 ± 0.1	36 ± 0.2 82 ± 0.1

^a^ Partial spreading on the oil surface

^b^ Complete spreading on the oil surface

^c^ Lack of biosurfactant production

**Table 3:** Characterization of biosurfactant produces by two strain SIS-20 and SIS-3

Properties	SIS-20	SIS-3	SLS ^a^	H_2_O
pH	6.95	7.24	7.13	7.0
Density (g/cm^3^)	1.18	1.16	1.01	1
Surface tension (dyne/cm)	69.808	62.524	59.393	68.6
Viscosity (poise)	12.6×10^–3^	10.7×10^–3^	10.1×10^–3^	9.1×10^–3^

^a^ Sodium Lauryl Sulphate

## Discussion

As marine actinobacteria are promising organisms to get high yielding of bioactive compounds, they are efficiently used in different fields such as bioremediation, drug development and cosmetic products. Hence, with that concern, we had paid more concern in marine actinobacteria. In this present study, almost 20 isolates were collected from marine water which are further designated as SIS-1 to SIS-20.

### Strain screening

The results of the present study reveal that, the isolates SIS-2, SIS-3, SIS-11, SIS-14, SIS-18 and SIS-20 showed very good hemolytic activity. Bernheimer and Avigad (1970) used this method to screen the biosurfactant producing microorganism. Carrillo et al. (1996) described the hemolytic activity as a primary technique to screen the biosurfactant activity. The zone of inhibition was directly proportional to the concentration of surfactant. In another study, Noha et al. (2004) reported that 16 % of false positive results in hemolytic action for biosurfactant selection are due to microorganism virulence issue, as well as that the biosurfactants that are poorly diffusible may not lyse the blood cells. Hence, hemolytic activity was recommended as a primary screening method. The hemolytic activity of the remaining 14 strains was ranged from (0 to 140 mm) and these 14 strains exhibited poor results for the drop collapsing method, oil spreading method (50–80 mm) and emulsification index (20–50 %) method.

In the drop collapse method, SIS-3 and SIS-20 showed positive results. The drop collapse technique may be considered as chief screening method for biosurfactant production. Bodour et al. (2003) reported that the drop collapse efficient technique may be employed for detection of biosurfactant producing microorganisms in natural environment. The oil spread method is an indirect dimension of surface action of a surfactant which is tested against oil; a higher surface action of biosurfactant is represented with a larger diameter of clearing zone (Rodrigues et al. 2006). Plaza et al. (2006) and Youssef et al. (2004) verified that the oil spreading method is an effective method to identify biosurfactant production by various microorganisms. The assay was also used for screening by Huy et al. (1999). Among the six isolates, SIS-3 and SIS-20 showed highest emulsification index. The emulsification index is a technique widely used to identify and quantify biosurfactants in cultures. Emulsification is an important process in alkane biodegradation (Thavasi et al. 2011). The diffusion of a liquid phase as microscopic droplets in an additional liquid continuous phase results in the development of emulsion (Desai and Banat 1997). This property is useful for making water/oil emulsions for food and cosmetics.

In all these studies, among the six isolates which are screened for primary test, only SIS-3 and SIS-20 showed maximum clear of zone, emulsification index and good result in drop collapse, hence these two isolates were studied further.

### Production media for biosurfactant

For all the isolates, SS + oil media showed more emulsification index than SS media. The use of vegetable oil as carbon sources to produce biosurfactants was found to be an effective and low-cost alternative (Abouseoud et al. 2008). Hence, SS + oil media were chosen for further production of biosurfactant from marine actinobacteria.

### Biosurfactant characterization

Attwood et al. (1970) already discussed that the higher the surface tension of biosurfactant, the higher will be the micelle forming activity which shows higher oil degradation activity. In the cosmetic industry, the cosmetic product quality including product viscosity and product stability get affected by different factors for example, foaming, emulsifying, water binding capability, dispersal and wetting features (Gharaei-Fathabad 2011). The variety of cleanup efforts varies extensively and depends on a number of issues, such as density of biosurfactant, the capacity of the spilling, weather as well as speed of reactions. The biosurfactant from SIS-3 was found to have more pH than SIS-20. Hence, the biosurfactant from SIS-3 can well accommodate in oceanic environment with respect to stability. Graham (2010) already mentioned that the “rise rate” of oil globules (their vertical velocity) is the rapidity at which oil particles move in the direction of the panel surface which is because of the dissimilarity in densities of oil and water. The closer the density of the biosurfactant in compared water, the lesser will be the time acquired for separation. Since SIS-3 showed best result with respect to pH, density, viscosity and surface tension, it was selected for further analysis and application.

### Orcinol assay

Production of a blue-green color after addition of orcinol indicated the presence of glycolipid in the biosurfactant produced by SIS-3 (Ahmad Mohammad et al. 2011). This was the first report on the production of rhamnolipid by marine *Nocardiopsis* sp.

### Stability test

The isolate SIS-3 maintained its best biosurfactant properties at the temperature of 50 °C as shown. This activity indicates the significance of the biosurfactant in pharmaceutical, food and cosmetic industries, where leading to achieve sterility is of paramount importance (Abouseoud et al. 2008; Mulligan et al. 1984). The results indicated that the increase in pH had a positive effect on emulsification activity and surface tension. This might be because of a better constancy of fatty acid surfactant micelles in the occurrence of sodium hydroxide and the precipitation of secondary metabolite at high pH value (Abouseoud et al. 2008; Abu-Ruwaida et al. 1991). With result to the effect of salinity on biosurfactant, the result suggested that the biosurfactant might be useful in marine environments and other systems where salt concentration is above physiological level. There are reports on some biosurfactants produced by bacteria showing stability in the presence of high salt concentration (Chandran and Das 2010).

Based on the results obtained from the present study, the biosurfactant produced by SIS-3 had its best activity at high temperature, alkaline pH and high salinity which could be useful for bioremediation of spills in marine environment. In contrast to the report of Khopade et al. (2012), moderately thermostable biosurfactant from marine *Nocardiopsis* sp. had been identified.

### Fourier transforms infrared spectroscopy (FT-IR) analysis

The FT-IR analysis result was correlated with previous work of Chander et al. (2012) and Yan-Ping et al. (2009). From the FT-IR data, it is evident that rhamnolipid form of biosurfactant was dominant in the biosurfactant produced by SIS-3.

### Gas chromatography–mass spectrophotometry (GC–MS) analysis

The major chemical constituent found in the present study is phenol, 3, 5-bis (1,1-dimethylethyl) which was responsible for surfactant activity. Hence, it can be interpreted that aromatic phenolic compounds might be responsible for the dissociation of hydrocarbon compounds present in the crude oil into many different simpler compounds (Kuo 1994).

### Strain identification

*Nocardiopsis* VITSISB (KC958579) was found to be closely related to *Nocardiopsis dassonvillei* (NR074635) with 98 % similarity.

#### Degradation of engine oil by immobilized cell in aquatic model

In the aquatic model, sugarcane juice and soya meal were used as carbon and nitrogen source, respectively. Sugarcane juice is rich in monosaccharides, such as glucose, fructose; disaccharide such as sucrose (81–87 %), oligosaccharides (6 %) and polysaccharides (8 %). It is also enriched with various inorganic salts such as potassium, sodium, calcium, magnesium, iron, aluminium, copper, zinc, manganese, cobalt, silicon, chloride, phosphate and sulphate. The product that remains after extracting most of the oil from whole soya beans is the soybean meal. Soybean meal is characterized of having higher content of crude protein, about 40–49 %. Proteins of soybean meal are characterized and they contain higher quantity of lysine, tryptophane, isoleucine, valine and threonine (Poultry Feeding Standards) (Ensminger et al. 1990). Hence, combination of sugarcane juice and soya meal can act as complete nutrient formulation for the growth of actinobacteria. *Nocardiopsis* VITSISB (KC958579) was applied as immobilized cells to degrade engine oil. The immobilized microorganism is now swiftly used in the laboratory. Rather than using biosurfactant alone, immobilized living cells are more potential in the biodegradation of hydrocarbons. The use of immobilized biosurfactant producing organism is a rapid, nontoxic, inexpesnsive, versatile method (Siemann and Wagner 1993).  The aquatic model provided similar oceanic environment of oil spill to the strain to degrade the oil so that the result of oil degradation can be correlated as of marine environment. In HPLC chromatogram, relative abundance was taken on *Y* axis and retention time was taken on *X* axis. The increase in the number of peaks after oil degradation indicated the breakdown of heavy hydrocarbons into several simpler compounds. The result of HPLC confirmed the degradation of engine oil by the isolated *Nocardiopsis* VITSISB (KC958579).

## Conclusion

The marine actinobacteria, *Nocardiopsis* VITSISB (KC958579) isolated from marine water sample, was found to be the proficient producer of rhamnolipid as well as potent degrader of engine oil under oceanic condition with reference to high pH, temperature and salinity. The biosurfactant having elevated emulsification activity, extra surface tension reduction, low density and higher viscosity, can be credibly used towards biodegradation of oil spill in ocean as compared to chemical dispersants. The biosurfactant from *Nocardiopsis* VITSISB (KC958579) offered an extra advantage of its production on renewable sources. Hence, this specific property of the biosurfactant may replace chemical surfactant in various fields. It can be concluded that the immobilized culture of *Nocardiopsis* VITSISB (KC958579) can act as a new scheme for oil spill cleanup in ocean with respect to reusability and cost effectiveness.
